# Fibreoptic aided retrograde intubation in an oral cancer patient

**DOI:** 10.4103/0019-5049.79876

**Published:** 2011

**Authors:** Sabyasachi Das, Mohan C Mandal, Bidyut B Gharami, Payel Bose

**Affiliations:** North Bengal Medical College, West Bengal, India

Sir,

The difficult airway and its management will continue to be reviewed. The incidence of difficult intubation in general population is around 5.8%,[1] but this incidence can rise significantly in the presence of airway tumours. Recently we encountered such a patient and we combined both retrograde and fibreoptic (FOB) intubation[2] in a difficult airway situation, compromised by a large oral cavity mass.

**Figure 1:**
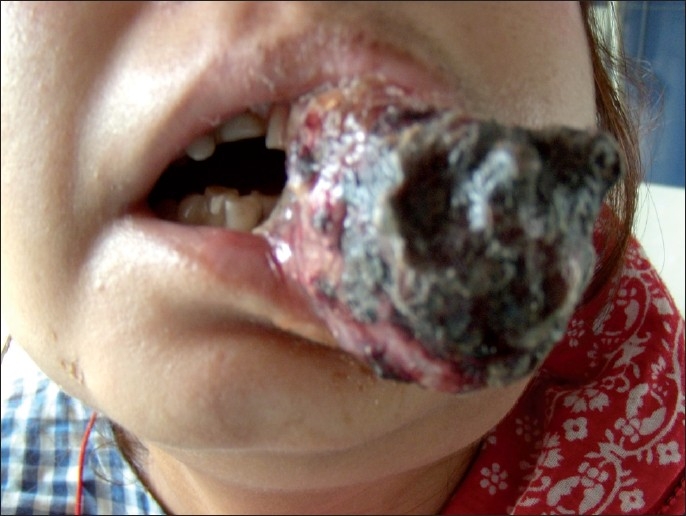
Growth of left cheek which has grown to present size over last 2 months

A 35-year-old lady presented with a rapidly progressing ulceroproliferative mass of left cheek extending beyond the left angle of the mouth. She had a restricted mouth opening to less than 1 cm only on the right side and a limited mandibular protrusion [Figure 1].

X-ray of the PNS revealed a deviated bony nasal septum towards left. CT scan of the PNS showed a lesion of the submandibular region extending to the mandibular arch, soft tissue of cheek and nasolabial area.

**Figure 2:**
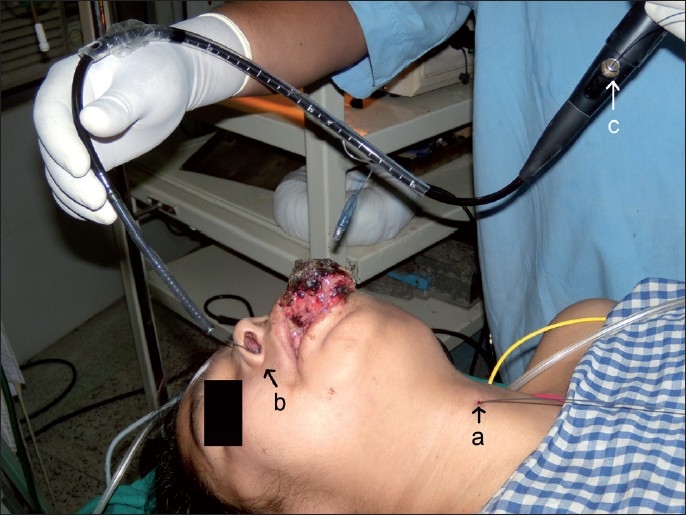
FOB loaded with a tracheal tube showing both ends of the Terumo guide wire. (a) Distal end of the guidewire, (b) guidewire through the right nostril, (c) proximal end of the guidewire through the proximal end of the working channel of the FOB

An awake, right nasotracheal intubation was planned. After preparation of the patient for an awake intubation, the cricothyroid membrane was punctured with a Tuohy needle through which a Terumo guide wire was fed in. A red rubber catheter was used to take the guide wire from the mouth through the right nostril. The flexible tip of the Terumo was fed through the distal end (2.8 mm) of the working channel of the FOB (OD 5.8 mm) to come out through the proximal end of the working channel. A fibrescope with a preloaded tracheal tube was rail-roaded through the right nostril through the trachea [Figure 2].

**Figure 3:**
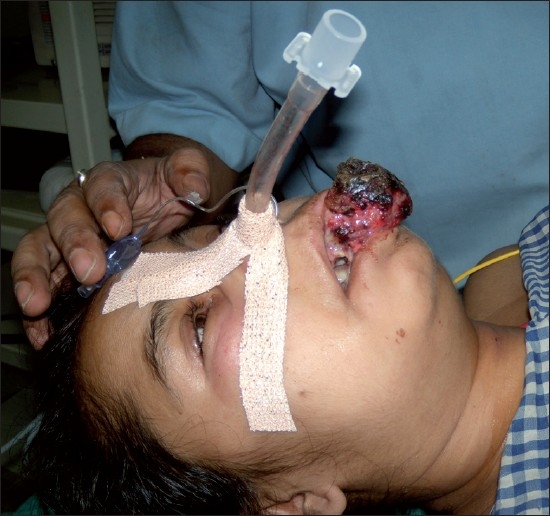
Intubated awake patient with a secure airway (permission for publication obtained from the patient)

The position of the tracheal tube was checked. Oxygen insufflation through the scope was done during the procedure. The patient tolerated the procedure well [Figure 3].

We had three options: blind nasal, FOB and tracheostomy. Blind nasal is a simple technique, but success at first pass is less[3] and there is more trauma and bleeding with more attempts. This may result in failure to visualise during subsequent FOB attempts. Awake FOB is the gold standard for anticipated difficult intubation. However, in one survey, it was observed that FOB is the first choice only in 8% difficult airway situations in medical colleges in India. FOB intubation has a difficult learning curve and its success depends upon skill, training and experience.[4] Tissue oedema and immobility due to tumour, distorted airway, copious secretions and chances of bleeding contribute to a higher failure rate.[3] In retrograde intubation, the endotracheal tube may move out of the larynx into the oesophagus or kink with failure to advance after guide catheters are removed.[5] Herein lies the importance of this modified technique that utilises a guidewire introduced with the retrograde approach which is subsequently used to guide the FOB for speedy advancement into the oropharynx occupied with tumour. To do so, guidewire must be long enough to accommodate the length of the scope. We used a sterile Terumo guidewire intended primarily to cannulate the common bile duct. The tip of this guidewire is very soft and becomes slimy in the presence of water so that it finds its way even in a small opening. So, chance of retrieving the catheter from the mouth or nostril at first pass, in the presence of an airway tumour, is more. Though not impossible, tracheostomy under local anaesthesia is difficult in advanced oropharyngeal cancers causing anatomical distortion of the anterior neck. This combination technique may be helpful to secure the airway reliably, safely and quickly in oral cancer patients requiring awake tracheal intubation for anticipated difficult airway situations.
